# Integrating pocket colposcope and telecolposcopy into VIA-based screening at primary healthcare centers for cervical cancer prevention in LMICs

**DOI:** 10.3389/fonc.2026.1902305

**Published:** 2026-08-10

**Authors:** Hasan Mahmud Reza, Hasin Anupama Azhari, Oyshi Chowdhury, Asim Kumar Bepari, Masum Chawdhury, Golam Abu Zakaria, Koustuv Dalal

**Affiliations:** 1 Department of Pharmaceutical Sciences, North South University (NSU), Dhaka, Bangladesh; 2 Institute of Natural Sciences, United International University (UIU), Dhaka, Bangladesh; 3 Alo Bhubon Trust (Alo-BT), Dhaka, Bangladesh; 4 Anhalt University of Applied Sciences, Kothen, Germany; 5 Division of Public Health Science, Institute of Health Sciences, Mid Sweden University, Sundsvall, Sweden

**Keywords:** cervical cancer, colposcopy, LMIC, pocket colposcopy, telecolposcopy, VIA, digital health

## Abstract

Cervical cancer is a prevalent global public health concern, particularly in low- and middle-income countries (LMICs). In such countries with limited resources, cervical cancer-mediated morbidity and mortality are also high due to a lack of effective screening and treatment facilities. Visual Inspection with Acetic Acid (VIA) is a non-invasive, low-cost alternative to cytology and HPV DNA testing. However, the efficacy of VIA is often compromised due to its low diagnostic accuracy and inter-subject variability. Integrating pocket and telecolposcopy with VIA at primary healthcare facilities may improve screening accuracy and coverage. This review examines published reports on the sensitivity and specificity of VIA, and highlights the limitations of cervical cancer screening programs in LMICs. It further discusses the challenges and opportunities of digital screening with pocket colposcopes and telecolposcopy, and the rationale for integrating these technologies with VIA in primary healthcare settings to improve cancer prevention coverage in LMICs.

## Introduction

Cervical cancer is one of the most prevalent cancers in the world. According to the WHO’s report from the year 2022, it is the fourth most common cancer among women (1). The global burden of cervical cancer is expected to rise substantially, with annual new cases projected to increase from approximately 570,000 to 700,000 between 2018 and 2030 (2). This growing liability disproportionately affects LMICs, where cervical cancer incidence and mortality remain alarmingly high. Previous reports have shown that over 85% of cervical cancer cases occur in LMICs across Africa, Latin America, the Caribbean, and Asia (3, 4).

It has been observed that the incidence and mortality of cervical cancer in LMICs are disproportionate to that of high-income countries due to the lack of detection and prevention programs, or, when they exist, due to barriers in their adoption and implementation (5–7). In these countries, the overall detection rates remain suboptimal (5, 8, 9). While evidence demonstrates that HPV-based screening confers superior protection against invasive cervical cancer compared with conventional cytology and other visual approaches of screening (10), LMICs are unable to adopt cytology and HPV-based screening for their large population due to multiple challenges. The majority of primary healthcare settings in LMICs outside major cities lack cytology screening facilities. It is worth mentioning that in Senegal, Benin, and the Ivory Coast, timely screening as part of the routine service in primary healthcare enabled a large number of women to be screened, while a substantial number of women with positive results were effectively treated with thermal ablation, having only minimal side effects (11). Since HPV testing is expensive and requires further lab facilities, alternative cost-effective, simple, and scalable methods- such as VIA and colposcopy remain critical components of cervical cancer screening strategies in resource-limited settings. VIA is a convenient and cost-effective method, particularly suited to low-resource settings. Using VIA, precancerous lesions are detected by the appearance of acetowhite changes after application of dilute acetic acid. Therefore, this technique supports immediate clinical assessment and early interventions. Nonetheless, there are concerns of substantial variability and sensitivity as accuracy largely depends on the skills of the technicians or nurses conducting the tests (12). International recommendations emphasize the importance of risk-based screening strategies, indicating that women with familial predisposition to cervical cancer may benefit from more vigilant and accessible screening methods (13, 14).

When individuals test positive with VIA, visual colposcopy procedures are usually followed to detect precancerous and cancerous lesions on the cervix (15). Although colposcopy is recommended after abnormal Pap smear findings or abnormal cytology and HPV results, it can also be beneficial for screening in LMICs (15, 16). Recent advances in digital imaging technology enable handheld cameras to capture high-resolution photograms. Digital imaging also enables data sharing and remote consultation between expert colposcopists and clinicians or nurses with limited experience. Consequently, colposcopies can significantly improve diagnostic accuracy (17). Compared with conventional optical colposcopes, digital colposcopy offers several practical benefits. Therefore, digital colposcopy can be easily implemented in LMICs, offering a low-cost solution with greater portability and user-friendliness. Recently, the mobile or pocket colposcope has been shown to perform comparably to a standard video colposcope (18). Pocket colposcopes can be conveniently operated by field-level healthcare providers, and the MobileODT Enhanced Visual Assessment system integrates computer-assisted diagnostic technology and artificial intelligence (AI) to achieve an accuracy rate exceeding 80%. This also minimizes some limitations associated with conventional VIA screening (19, 20). This review explores whether integrating digital technology to support tele-mentored, decentralized VIA screening at primary healthcare centers can provide a more reliable, convenient, yet cost-effective alternative to more expensive screening methods in cervical cancer prevention programs in LMICs.

## Methodology

A comprehensive literature search was performed in PubMed/MEDLINE, Scopus, Web of Science, and Google Scholar to identify publications from January 2005 to March 2026. Additional articles were identified through reference screening. The search strategy utilized combinations of terms such as *cervical cancer screening*, *VIA*, *digital colposcopy*, *portable, pocket, or mobile colposcope*, *telecolposcopy*, *telemedicine*, *HPV*, *primary healthcare*, *LMICs*, and *resource-limited settings*. Eligible studies included original research, clinical and implementation studies, diagnostic studies, systematic reviews, meta-analyses, and relevant policy documents that evaluated VIA, digital or portable colposcopy, telecolposcopy, and related digital health technologies for cervical cancer screening. Exclusion criteria comprised studies unrelated to cervical cancer screening, editorials, conference abstracts, case reports and duplicate publications.

## Main body

### Pathophysiology of cervical cancer

Persistent infection with high-risk Human Papillomavirus (HPV) is responsible for the development of cervical cancer. HPV16 and HPV18 are the most virulent types in the emergence of this cancer. HPVs are very small, non-enveloped viruses that encode E6 and E7 proteins, the key drivers of cervical carcinogenesis (21). Although most women have an immune system to clear out the virus, yet it can persist in the cervix transformation zone (TZ) and initiate neoplastic progression. HPV primarily targets the basal cells of the stratified cervical epithelium. Following entry into host cells, viral DNA is incorporated into the host genome, and viral oncoproteins E6 and E7 are overexpressed. These proteins inactivate tumor suppressor protein p53 and retinoblastoma (Rb), disrupting cell-cycle regulation, promoting genetic instability, and leading the progression from intraepithelial neoplasia (CINI-CINIII) to invasive cervical carcinoma.

### Age-specific considerations for cervical cancer screening

The age-specific distribution of cervical cancer reflects the progression of persistent high-risk human HPV infection. While HPV infection is most prevalent among younger women, the majority of cases resolve without intervention. Progression to cervical intraepithelial neoplasia grade 2 or worse (CIN2+) or invasive cervical cancer generally requires 10 to 20 years of persistent infection (22, 23). As a result, invasive cervical cancer is most frequently diagnosed in women aged 35 to 50 years, which supports recommendations to begin screening after early adulthood rather than during adolescence (24, 25).

This epidemiological pattern has significant implications for screening programs. In high-income countries, organized HPV- or cytology-based screening has markedly reduced cervical cancer incidence by enabling early detection and treatment of precancerous lesions. Conversely, many low- and middle-income countries (LMICs) continue to rely on VIA due to resource constraints, which leads to delayed diagnosis and a higher disease burden among middle-aged women (25). In Bangladesh, the majority of VIA- and colposcopy-positive women are between 26 and 44 years of age, underscoring the need to strengthen screening coverage in this demographic (15). Integrating primary screening with portable digital colposcopy and telecolposcopy may facilitate timely triage of screen-positive women, improve access to specialist assessment, and enhance early detection and referral within primary healthcare systems in LMICs.

### Cervical cancer screening scenario in LMICs

Cervical cancer screening coverage remains very low in LMICs, especially in Asian and African regions. Although high-income countries have achieved adequate screening coverage through population-based screening programs, most LMICs still rely on facility-based and opportunistic approaches. Here, VIA remains the most used screening method, whereas HPV DNA testing is gradually being introduced as pilot programs. Studies reveal that the pooled estimate of cervical cancer screening rate in eight countries (Bangladesh, China, India, Iran, Japan, Korea, Nepal, and Thailand) is 28% from 2012 to 2023 (26). Data from a national survey and a meta-analysis indicate very low screening rates in most South Asian LMICs. The worst part is that the rate is often below 20%. To add more, according to the WHO STEP 2018 survey, only 6.2% of women in Bangladesh have ever been screened (27). A systematic review and meta-analysis of studies conducted in Nepal showed screening rates of 17% in the hospital settings and 16% in the community setting, indicating consistently low uptake (28). Another study conducted in five hospitals in Nepal demonstrates only 22.2% screening among 657 women (29). In the Kalutara district of Sri Lanka, a study was undertaken to realize the feasibility of accepting a new HPV/DNA test for primary screening of cervical cancer among 35-year-old ever-married women, and the response rate was 91.1% (30). A community-based cross-sectional survey on cervical cancer in Kampong Speu Province, Cambodia, showed that among 440 respondents, only 7% of them have ever undergone a Pap test (31). A snapshot of several studies on cervical cancer screening conducted in LMICs is shown in **Table 1**.

**Table 1:** Studies on cervical cancer screening conducted in LMICs.

Author (year)	Country	Sample size	Data year	Study type	Screening %/uptake	DOI/URL	References
(Khan et al., 2024)	Bangladesh	4381	2018	Cross-sectional	6.2% screened	10.1007/s43999-024-00053-x	(27)
(Islam et al., 2015)	Bangladesh	1590	2013-2014	Cross-sectional	8.3% screened	10.1634/theoncologist.2015-0235	(32)
(Um & Sopheab, 2025)	Cambodia	19496	2022	Secondary analysis	15.3% screened	10.1371/journal.pone.0295881	(33)
(Touch & Oh, 2018)	Cambodia	440	2016	Cross-sectional	7% screened	10.1186/s12885-018-4198-8	(31)
(Aswathy et al., 2012)	India	809	2009	Cross-sectional	6.9% screened	*PMCID: PMC3461731	(34)
(Sanavelli et al., 2026)	India	334	2025	Cross-sectional	7% screened	10.18203/2320-1770.ijrcog20260559	(35)
(Srivastava et al., 2022)	India	1020	2015-2017	Cross-sectional	3.9% screened	10.31557/APJCP.2022.23.8.2771	(36)
(Han et al., 2016)	Myanmar	230	2012	Cross-sectional	16.5% screened	10.9734/BJMMR/2016/28156	(37)
(Shrestha et al., 2022)	Nepal	17 studies pooled	2000–2020	Meta-analysis	16–17% pooled	10.1016/j.puhe.2022.06.007	(28)
(Shamaun et al., 2023)	Pakistan	226	2021-2022	Cross-sectional	15.9% screened	10.1055/s-0042-1751093	(38)
(Hirani et al., 2021)	Pakistan	384	2016	Cross-sectional	34.31% screened	10.1097/CEJ.0000000000000590	(39)
(Imoto et al., 2020)	Philippines	338	2017	Cross-sectional	13.9% screened	10.31557/APJCP.2020.21.11.3145	(40)
(Nilaweera et al., 2012)	Sri Lanka	169	2011	Cross-sectional	17.2% screened	10.7314/apjcp.2012.13.4.1193	(41)
(Witharana et al., 2015)	Sri Lanka	282	2014	Cross-sectional	7.6% screened	10.1186/s12889-015-2531-6	(42)
(Nguyen et al., 2025)	Vietnam	6668	2020-2021	Decomposition analysis	28.2% screened	10.1186/s12889-025-22511-y	(43)
(Phung et al., 2023)	Vietnam	398	2021	Cross-sectional	46.98% screened	10.1371/journal.pgph.0001817	(44)

*DOI is not available.

### Why cervical cancer screening is inadequate in LMICs

Determining the prevalence of cervical cancer and identifying associated risk factors requires coordinated epidemiological, clinical, and laboratory-based approaches. A foundational action is the establishment and strengthening of population-based cancer registries, which systematically collect data on cancer incidence, mortality, and survival, and this is strongly recommended by the World Health Organization and the International Agency for Research on Cancer for surveillance and policy planning (24, 45). Conducting well-designed epidemiological studies, including cross-sectional, case–control, and cohort studies, is essential for identifying and quantifying risk factors. These studies should collect standardized data on known determinants such as persistent high-risk HPV infection, age at first sexual activity, presence of multiple sexual partners, smoking, long-term oral contraceptive use, high parity, family history of cervical cancer, and immunosuppression (e.g., HIV infection) (24). In LMICs, only sporadic studies are conducted, and registry coverage and data completeness remain poor. Also, integrating such data with existing reproductive health or vaccination programs is absent; hence, screening is less prioritized.

Since 2020, the American Cancer Society has recommended the primary high-risk HPV test as the preferred approach for cervical cancer screening. However, the shift from conventional cytology-based screening (Pap test) and co-testing strategies combining cytology with HPV detection has progressed gradually and remains limited in many settings. Current recommendations also support HPV genotyping for types 16 and 18, together with reflex cytology for other high-risk HPV genotypes to improve risk stratification and clinical management (46). In parallel, newer diagnostic approaches such as dual-stain testing for p16/Ki-67 and extended HPV genotyping are increasingly being integrated into cervical cancer screening and treatment algorithms as alternative strategies. In addition, methylation-based assays have emerged as a promising area of research and are being extensively explored worldwide for their potential to improve early detection and patient management (47). These programs not only reduce disease incidence but also generate valuable epidemiological data on the prevalence of precancerous lesions (e.g., CIN2+) and high-risk HPV infection. HPV testing, in particular, provides highly sensitive detection of oncogenic infections and serves as a robust tool for population-level risk assessment (48). However, resource shortages are a vital impediment to implementing organized screening programs using validated methods such as cytology and HPV DNA testing in LMICs. In LMICs, a shortage of trained workforce (e.g., cytopathologists) and limited diagnostic facilities are major barriers for screening. For example, Bangladesh severely faces a shortage of skilled providers, especially in rural areas (15, 49). Similarly, many LMICs lack organized screening programs and rely on opportunistic screening and provider referral rather than systematic population coverage, reducing overall coverage (15, 50). Laboratory facilities for molecular detection of HPV are scarce across the country, and HPV testing is significantly low. Also, the HPV vaccine is not widely available or accessible in many LMICs, mainly due to its cost, the lack of campaigns, limited supply, and the absence of storage facilities in remote areas (15, 51). It can be noted that donor-supported government initiatives have been launched lately in Bangladesh, Pakistan, Nepal, and other countries (52–54).

Apart from these factors, numerous community- and individual-level factors undermine the screening program’s effectiveness. Across LMICs, lack of awareness is the most common individual-level barrier, often linked to low education and inadequate health communication systems (15). One of the most consistently reported barriers is poor knowledge about cervical cancer, its risk factors, and the benefits of screening. Many women are unaware that screening can prevent cancer or detect it early. Studies in Bangladesh show that knowledge about screening procedures and prevention is particularly limited, even when general awareness of the disease exists (50, 55). A study conducted in Eastern India evaluating parental acceptance of HPV vaccination found that only 40% of parents were initially willing to vaccinate their daughters. However, acceptance increased substantially to 80% after participants received a brief educational intervention on the vaccine’s benefits and safety (51). Furthermore, screening is associated with stigma, modesty concerns, or embarrassment. Women may need permission from husbands or family decision-makers. Misconceptions about cancer (e.g., fatalism or linking it to immoral behavior) also persist. In Bangladesh, family influence and social stigma significantly limit screening uptake, especially in rural and conservative communities (55). Fear of pain during screening/diagnosis, or invasive procedures discourages many women from participating. Shyness and discomfort with pelvic examinations are also widely reported barriers in Bangladesh and other LMICs (50). This further ties with limited access and financial constraints. Even when screening is nominally cost-free, indirect costs, including transportation and lost wages, discourage women. Cost-related concerns are repeatedly identified in Bangladesh and other LMICs (56).

### VIA and colposcopy are feasible screening methods for rural women in LMICs

International recommendations emphasize the importance of risk-based screening strategies, indicating that women with a familial predisposition to cervical cancer may benefit from more vigilant and accessible screening methods. Timely and regular screening using cost-effective approaches such as VIA is a practical and evidence-based method, particularly suited to low-resource settings, as it enables the detection of precancerous lesions through the appearance of acetowhite changes after diluting acetic acid application. VIA is widely used in “screen-and-treat” programs because it provides immediate results, enables rapid clinical decision-making and facilitates early intervention, which is critical for expanding coverage and reducing loss to follow-up (57). In this background, data from VIA-positive cases can be used as an indirect indicator of the prevalence and precancerous cervical lesions, although this approach possesses an accuracy issue (58). By providing short training to nurses and non-physician healthcare providers, it is possible to expand the service reach. While an individual study has reported high diagnostic performance of VIA, with sensitivities of 89.83% and specificities of 96.23%, it does not reflect the overall performance of VIA, and it should not be extrapolated to routine clinical practice (59). Systematic reviews and meta-analyses demonstrate that VIA exhibits moderate but highly variable diagnostic accuracy, with pooled sensitivities of approximately 73-88% and specificities of 83-87% for CIN2+ (58, 60, 61). This variability is largely attributable to differences in provider expertise, training, patient characteristics, and screening protocols. Findings from VIA rely on subjective visual interpretation of acetowhite changes, which also compromises in prevalence estimates of CIN2+ and cancer (62). False negatives are especially threatening because they delay detection of precancerous lesions and increase the risk of progression to invasive cancer.

Colposcopy is recommended in triaging HPV-positive women since it enables detailed visualization of the cervix due to magnification, so that the observer can clearly detect the presence and severity of CIN2+ lesions and direct biopsies for histological confirmation (48). However, it has been the practice to investigate the VIA-positive cases through colposcopy in some LMICs, where HPV testing or Pap smear is somewhat difficult, particularly in rural settings (63). In addition, colposcopy helps determine lesion size, location, and eligibility for specific treatments such as LEEP or thermal ablation. However, the operator’s expertise plays an important role in distinguishing low- and high-grade lesions by colposcopy (64). Taken together, VIA and colposcopy can still contribute to approximate or operational estimates of disease burden in LMICs because these countries have limited facilities for cytology or HPV testing.

### Cytology and HPV tests over visual approaches

Cytology remains a well-established screening tool with proven specificity in organized programs with quality control. It is less dependent on subjective visual judgment than VIA, leading to more consistent results when integrated into organized screening. Yet, its sensitivity for CIN2+ typically ranges from 50-70% (10, 65). Similarly, HPV DNA testing is generally accepted for determining the causative organism of cervical cancer, yet it does not detect the precancerous lesion or cancer itself. The fact is, the HPV-positive cases can act as transient carriers without any current pathological changes to the cervix. This test identifies the viral cause of cervical carcinogenesis rather than relying solely on visual or cytologic changes, improving early detection and long-term risk stratification. It has been demonstrated that HPV infections are normally cleared within 2 years by the immune system; however, persistent infection poses a high risk of developing cervical cancer, thus, this test is essentially important (66, 67). Meta-analyses report that HPV DNA testing has >90% sensitivities for CIN2+ (62). Consequently, WHO now recommends HPV DNA testing as the preferred cervical screening method due to its superior sensitivity and reproducibility (68).

### Benefits of combining pocket colposcopes and telecolposcopy with VIA

Literature review indicates that most studies have focused on determining the proportion of women who attended colposcopy following a positive HPV screening result (69–72). However, the reality is that it is almost impossible to undertake the HPV testing or cytology-based screening of a large population in most LMICs due to a shortage of facilities, funds, and trained staff. Rodney Hull has noted similar limitations and therefore proposes VIA as an alternative method for large-scale population screening (73). As in wider cases of HPV-positive young women, the virus naturally disappears after a certain period; therefore, HPV-positive cases are not given immediate therapy but are instructed to attend a follow-up visit. This follow-up visit is missed by many; as a result, they remain untreated, even when it turns into the precancerous state and finally are diagnosed later with advanced-stage cancer. On the other hand, if VIA-positive cases are deeply suspicious by colposcopies in the same visit, gynecologists may quickly judge and may refer to the appropriate treatment options. Another study reported that HPV testing combined with immediate referral for colposcopy achieved a substantially high sensitivity (89%), although it was also associated with a high false-positive rate (38%) (74). Following decentralization of services, the number of women undergoing Pap smear screening at primary healthcare facilities and subsequently receiving colposcopy increased threefold (71). In addition, the proportion of women from rural centers experiencing prolonged waiting time for colposcopy decreased to 21.7% (71). Similarly, Blackenberg et al. (75) observed an 18% increase in the number of patients receiving colposcopy service at a rural health center compared with the preceding year, indicating a positive trend in service utilization. The study also documented a substantial reduction in waiting time, averaging 29 days. These findings suggest that establishing colposcopy services in rural hospitals can significantly enhance women’s access to essential cervical screening follow-up care (72).

A more convenient version of colposcopy is digital colposcopy, commonly known as the pocket colposcope. This can be operated by a nurse and has been demonstrated to increase screening quality in sub-Saharan Africa (76). The pocket colposcopes of generation 3 and 4 can perform like a standard-of-care colposcope with higher image contrast (77). The use of high-resolution digital imaging has enhanced the detection of cervical lesions and facilitated the sharing of images between experienced and less experienced healthcare providers (17). Pocket colposcopes supplement VIA by providing magnified digital visualization and permanent image documentation (78, 79). A randomized crossover study involving 250 women, with histopathology as the reference standard, found that the pocket colposcope achieved 96.3% sensitivity and 92.3% specificity for CIN2+, results comparable to those of a standard video colposcope (18). After visual inspection with VIA, triage using the pocket colposcope reduced overtreatment from 46.8% to 4.8% and referrals from 12.4% to 6.0%, thereby facilitating a single-visit screen-triage-treat strategy. With focused short-term training, primary healthcare professionals (PHPs) and nurses can acquire the necessary skills and competency to identify and manage pre-invasive or early invasive cervical diseases within primary care settings (64). We suggest that women with VIA- or HPV-positive results should subsequently undergo triage with pocket colposcopy, performed by PHPs during the same visit. With telecolposcopy-enabled remote specialist consultation (when required), women with positive colposcopic findings can then be managed with biopsy, thermal ablation for eligible lesions, or referral, as per national guidelines. Hence, expanding these services at the community level may reduce travel burdens, shorten waiting time, lower healthcare-related costs, and minimize complications associated with delayed diagnosis and treatment. Nevertheless, it is important to recognize that much of the existing evidence is based on feasibility studies, pilot investigations, and observational cohorts with relatively small sample sizes.

Since few studies have directly compared VIA alone, VIA supplemented with portable or pocket colposcopy, and standard colposcopy using histopathology-confirmed CIN2+ as the reference standard, our discussion focusing on the integration of pocket colposcopy into VIA at primary healthcare centers relies primarily on complementary evidence rather than direct comparative trials. **Table 2** provides a concise summary of the reported findings regarding the screening and diagnostic performance of these approaches.

**Table 2:** Comparative evaluation of VIA, VIA with pocket or telecolposcopy, and conventional colposcopy in cervical cancer screening.

Screening approaches	Evidence supporting CIN2+ detection	Major advantages	Major limitations	Key references
Visual inspection with acetic acid (VIA)	Meta-analyses report a pooled sensitivity of approximately 79% (95% CI: 73–84%) and a specificity of 85% (95% CI: 81–89%), although substantial heterogeneity exists across studies. Performance varies with provider training and quality assurance.	This approach offers low cost, immediate results, and requires minimal infrastructure, making it suitable for screen-and-treat programs in low- and middle-income countries.	Interpretation is subjective, leading to considerable inter-observer variability, reduced reproducibility, and an increased risk of false-positive and false-negative results.	(24, 60, 80, 81)
VIA + pocket/digital colposcopy	Available evidence suggests that portable digital colposcopy enhances cervical visualization, image documentation, lesion characterization, and diagnostic confidence. Comparative data based on histopathology are still limited. A randomized crossover study shows that pocket colposcope achieved 96.3% sensitivity and 92.3% specificity for CIN2+, results comparable to those of a standard video colposcope. VIA with pocket colposcope reduced overtreatment from 46.8% to 4.8% and referrals from 12.4% to 6.0%.	It enables image capture, magnification, documentation, quality assurance, remote consultation, and provider training, while maintaining portability and a relatively low cost. Most importantly, it facilitates a single-visit screen-triage-treat strategy.	There is limited direct comparative evidence showing improved diagnostic accuracy over VIA alone. Device availability and training requirements also remain significant challenges.	(18, 78, 79, 82, 83)
Telecolposcopy (digital image review by specialists)	Comparative data remain limited. Most evidence shows strong agreement between remote and onsite colposcopic assessments and improved access to specialist consultation, rather than direct comparisons with VIA alone.	This strategy expands specialist access, supports remote diagnosis, and improves referral triage, quality assurance, and continuing education, particularly in rural settings.	This approach requires reliable digital infrastructure, consistent internet connectivity, secure data transmission, and standardized imaging protocols.	(78, 84, 85)
Conventional colposcopy	Colposcopy is the standard diagnostic examination after abnormal screening. Diagnostic accuracy depends on examiner expertise, while histopathological biopsy remains the reference standard for CIN2+ diagnosis.	It provides high-quality magnified visualization, enables directed biopsy, and is recognized as an established clinical standard.	It relies on expensive equipment and specialist expertise, which limits availability in primary healthcare settings and many low- and middle-income countries.	(24, 86–88)

Interestingly, recent development of portable colposcopy systems marks an important advancement for cervical cancer screening in LMICs, for example, the Enhanced Visual Assessment (EVA) system combines smartphone-based technology with portable imaging systems to capture high-resolution cervical images, thereby improving visualization, documentation, and opportunities for remote expert consultation (89). The multimodal mobile colposcope (MMC) further expands these capabilities by integrating wide-field imaging with high-resolution micro endoscopy. This approach allows clinicians not only to identify suspicious cervical lesions but also to obtain subcellular-level images that facilitate differentiation between high-grade and low-grade dysplasia (90). In Zambia and India, pilot studies incorporating digital cervicography along with VIA demonstrated improved lesion detection and better inter-observer agreement compared with conventional VIA-based screening alone (91). Community-based screening initiatives in India that employed expert review of digitally captured cervical images successfully screened more than 13000 women, among whom 5.3% exhibited abnormal findings (92, 93). These findings are consistent with growing evidence supporting the role of digital health technologies in enhancing cervical cancer screening performance and improving patient outcomes, particularly in LMIC settings (92, 94). The mixed e-health educational strategies have been shown to nearly double screening uptake compared with standard approaches (94).

Quality assurance remains a fundamental component of effective cervical cancer screening programs. Studies have shown that incorporating specialists to review colposcopy data improves the diagnostic accuracy of VIA (95). Thus, in remote healthcare facilities, colposcopic images captured by PHPs can be uploaded to cloud-based platforms for expert evaluation and quality assurance. This enables prompt consultation with experienced colposcopists while the patient remains at the healthcare facility and strengthens overall quality control measures (85).

As mobile colposcopy enables the secure transmission of high-resolution cervical images captured by trained nurses or PHP to specialists for remote assessment and consultation, this eliminates unnecessary referrals and lowers the overall cost of diagnostic screening and follow up services. Because patients in underserved settings do not need to travel to tertiary hospitals for expert evaluation. This further saves both time and money, reduces diagnostic delays, while accelerates timely decision-making for clinical intervention (20, 92, 96). However, it is important to have robust data privacy safeguards, informed consent for digital image sharing and secure transmission of cervical (24). Furthermore, culturally sensitive community engagement is critical for increasing acceptance and participation in cervical cancer screening programs in LMICs.

It is worth emphasizing that the effective implementation of pocket colposcope and telecolposcopy in primary healthcare requires adequate training and competency assessment of PHPs. Structured training should encompass cervical anatomy, recognition of normal and abnormal cervical findings, image acquisition, infection prevention, patient counselling, and referral criteria. Competency should be assessed through standardized image interpretation, supervised clinical practice, and periodic quality assurance with remote expert feedback to minimize inter-observer variability and maintain diagnostic accuracy (24, 80). Also, portable and digital colposcopy technologies have several practical limitations that may affect their implementation in low-resource settings. Image quality can vary depending on device specifications, ambient lighting, operator experience, cervical preparation, and image acquisition technique, all of which can affect diagnostic accuracy and interobserver agreement (78). In telecolposcopy, reliable internet connectivity is required for timely image transmission and remote consultation, although asynchronous systems can partially mitigate this constraint. Furthermore, successful implementation depends on stable electricity, regular device maintenance and calibration, secure data storage, and technical support, which are often insufficient in rural and resource-constrained settings (97).

## Conclusions

The current literature suggests that nurse-led mobile colposcopy may be a promising, scalable digital health strategy for cervical cancer screening, especially in resource-constrained settings where access to specialized physicians remains limited (20, 93). The integration of artificial intelligence and tele-mentoring into mobile colposcopy platforms with PHP could strengthen the existing screening efficacy. Thus, early detection and prevention initiatives can be improved by increasing the accuracy and accessibility of screening services for underserved populations. Within the framework of secondary cervical cancer prevention, pocket colposcopy may serve as a point-of-care triage and diagnostic adjunct at primary healthcare centers. This approach may offer greater reliability than VIA alone, where accurate identification of precancerous lesions is essential for timely intervention.

## References

[1] World Health Organization . Cervical Cancer Elimination Initiative (2026). Available online at: https://www.who.int/initiatives/cervical-cancer-elimination-initiative (Accessed May 21, 2026).

[2] World Health Organization . Global Strategy to Accelerate the Elimination of Cervical Cancer as a Public Health Problem (2020). Available online at: https://www.who.int/publications/i/item/9789240014107 (Accessed May 22, 2026).

[3] Duncan J Harris M Skyers N Bailey A Figueroa JP . A call for low- and middle-income countries to commit to the elimination of cervical cancer. Lancet Reg Health Am. (2021) 2. doi: 10.1016/j.lana.2021.100036 34693395 PMC8507431

[4] Vaccarella S Laversanne M Ferlay J Bray F . Cervical cancer in Africa, Latin America and the Caribbean and Asia: Regional inequalities and changing trends. Int J Cancer. (2017) 141:1997–2001. doi: 10.1002/ijc.30901 28734013

[5] Pimple SA Mishra GA . Global strategies for cervical cancer prevention and screening. Minerva Ginecol. (2019) 71:313–20. doi: 10.23736/S0026-4784.19.04397-1 30808155

[6] Bedell SL Goldstein LS Goldstein AR Goldstein AT . Cervical cancer screening: Past, present, and future. Sex Med Rev. (2020) 8:28–37. doi: 10.1016/j.sxmr.2019.09.005 31791846

[7] Maine D Hurlburt S Greeson D . Cervical cancer prevention in the 21st century: cost is not the only issue. Am J Public Health. (2011) 101:1549–55. doi: 10.2105/AJPH.2011.300204 21778496 PMC3154220

[8] Malta DC Prates EJS Silva A Santos FMD Oliveira GC Vasconcelos N . Inequalities in mammography and Papanicolaou test coverage: a time-series study. Sao Paulo Med J. (2020) 138:475–82. doi: 10.1590/1516-3180.2020.0166.R1.02092020 33111921 PMC9685575

[9] Chocontá-PIraquive LA Alvis-Guzman N De la Hoz-Restrepo F . How protective is cervical cancer screening against cervical cancer mortality in developing countries? The Colombian case. BMC Health Serv Res. (2010) 10:270. doi: 10.1186/1472-6963-10-270 20846446 PMC2949854

[10] Ronco G Dillner J Elfström KM Tunesi S Snijders PJF Arbyn M . Efficacy of HPV-based screening for prevention of invasive cervical cancer: follow-up of four European randomised controlled trials. Lancet. (2014) 383:524–32. doi: 10.1016/S0140-6736(13)62218-7 24192252

[11] Selmouni F Sauvaget C Dangbemey DP Kpebo DDO Dieng NM Lucas E . Lessons learnt from pilot cervical cancer screening and treatment programmes integrated to routine primary health care services in Benin, Cote d’Ivoire, and Senegal. JCO Glob Oncol. (2022) 8:e2200051. doi: 10.1200/GO.22.00051 36070534 PMC9812504

[12] Huchko MJ Sneden J Sawaya G Smith-McCune K Maloba M Abdulrahim N . Accuracy of visual inspection with acetic acid to detect cervical cancer precursors among HIV-infected women in Kenya. Int J Cancer. (2015) 136:392–8. doi: 10.1002/ijc.28996 24889387 PMC4214890

[13] World Health Organization . Guidelines for Screening and Treatment of Precancerous Lesions for Cervical Cancer Prevention (2013). Available online at: https://www.who.int/publications/i/item/9789241548694 (Accessed May 14, 2026). 24716265

[14] Needleman H Huff J . The International Agency for Research on Cancer and obligate transparency. Lancet Oncol. (2005) 6:920–1. doi: 10.1016/S1470-2045(05)70442-3 16321758

[15] Azhari HA Chawdhury M Islam F Zakaria GA Dalal K Reza HM . Visual inspection with acetic acid and colposcopy: screening of cervical cancer in resource-limited healthcare settings. Front Glob Women’s Health. (2025) 6. doi: 10.3389/fgwh.2025.1684205 41458906 PMC12740937

[16] Gunes AC Ozgul N Turkyılmaz M Kara F Unlu F Ayhan A . Evaluation of colposcopy after the addition of human papillomavirus testing to the Turkish cervical cancer screening program. Cancer Med. (2023) 12:21751–60. doi: 10.1002/cam4.6740 37994572 PMC10757080

[17] Spitzer M . The era of “digital colposcopy” will be here soon. J Low Genit Tract Dis. (2015) 19:273–4. doi: 10.1097/LGT.0000000000000140 26225943

[18] Natarajan J Mathur S Vishnubhatla S Kumar S Vashist S Ramanujam N . Can portable colposcopes replace standard-of-care colposcopes? A crossover trial of two portable colposcopes with a standard-of-care video colposcope. Asian Pac J Cancer Prev. (2022) 23:4013–21. doi: 10.31557/APJCP.2022.23.12.4013 36579981 PMC9971471

[19] Dei-Adomakoh Y Effah K Tekpor E Crabbe S Amuah JE Wormenor CM . Cervical precancer screening with HPV DNA testing and mobile colposcopy in women with sickle cell disease in Accra, Ghana. Ecancermedicalscience. (2023) 17:1571. doi: 10.3332/ecancer.2023.1571 37533951 PMC10393310

[20] Effah K Wormenor MC Tekpor E Amuah JE Atuguba HB Mensah ENO . Mobile colposcopy by trained nurses in a cervical cancer screening programme at Battor, Ghana. Ghana Med J. (2022) 56:141–51. doi: 10.4314/gmj.v56i3.3 37448995 PMC10336638

[21] Yeo-Teh NSL Ito Y Jha S . High-risk human papillomaviral oncogenes E6 and E7 target key cellular pathways to achieve oncogenesis. Int J Mol Sci. (2018) 19:1706. doi: 10.3390/ijms19061706 29890655 PMC6032416

[22] Saslow D Andrews KS Manassaram-Baptiste D Smith RA Fontham ETH American Cancer Society Guideline Development Group . Human papillomavirus vaccination 2020 guideline update: American Cancer Society guideline adaptation. CA Cancer J Clin. (2020) 70:274–80. doi: 10.3322/caac.21616 32639044

[23] Bruni L Serrano B Roura E Alemany L Cowan M Herrero R . Cervical cancer screening programmes and age-specific coverage estimates for 202 countries and territories worldwide: a review and synthetic analysis. Lancet Glob Health. (2022) 10:e1115–27. doi: 10.1016/S2214-109X(22)00241-8 35839811 PMC9296658

[24] World Health Organization . WHO Guideline for Screening and Treatment of Cervical Pre-Cancer Lesions for Cervical Cancer Prevention (2021). Available online at: https://www.who.int/publications/i/item/9789240030824 (Accessed May 14, 2026). 34314129

[25] Sung H Ferlay J Siegel RL Laversanne M Soerjomataram I Jemal A . Global cancer statistics 2020: GLOBOCAN estimates of incidence and mortality worldwide for 36 cancers in 185 countries. CA: A Cancer J For Clin. (2021) 71:209–49. doi: 10.3322/caac.21660 33538338

[26] K C Bhandari A Htay ZW Parvin R Murakami M Matsuda T Abe SK . Prevalence of cervical cancer screening in Asia: A systematic review and meta-analysis. Asian Pac J Cancer Prev. (2025) 26:4269–81. doi: 10.31557/APJCP.2025.26.12.4269 41459841 PMC13237447

[27] Khan MJ Kannan P Sayma Winser SJ . Population-based cross-sectional survey of cervical cancer screening prevalence and socio-demographic correlates in Bangladeshi women. Res Health Serv Reg. (2024) 3:17. doi: 10.1007/s43999-024-00053-x 39500796 PMC11538205

[28] Shrestha AD Andersen JG Gyawali B Shrestha A Shrestha S Neupane D . Cervical cancer screening utilization, and associated factors, in Nepal: a systematic review and meta-analysis. Public Health. (2022) 210:16–25. doi: 10.1016/j.puhe.2022.06.007 35863158

[29] Dangal G Dhital R Dwa YP Poudel S Pariyar J Subedi K . Implementation of cervical cancer prevention and screening across five tertiary hospitals in Nepal and its policy implications: A mixed-methods study. PloS Global Public Health. (2024) 4:e0002832. doi: 10.1371/journal.pgph.0002832 38236836 PMC10796028

[30] Perera KCM Mapitigama N Abeysena H . The feasibility of new HPV/DNA test as a primary cervical cancer screening method among 35- years- old ever-married women in Kalutara district; a cross-sectional study. BMC Public Health. (2021) 21:131. doi: 10.1186/s12889-021-10190-4 33441083 PMC7805031

[31] Touch S Oh J-K . Knowledge, attitudes, and practices toward cervical cancer prevention among women in Kampong Speu Province, Cambodia. BMC Cancer. (2018) 18:294. doi: 10.1186/s12885-018-4198-8 29544466 PMC5856224

[32] Islam RM Bell RJ Billah B Hossain MB Davis SR . Lack of understanding of cervical cancer and screening is the leading barrier to screening uptake in women at midlife in Bangladesh: population-based cross-sectional survey. Oncologist. (2015) 20:1386–92. doi: 10.1634/theoncologist.2015-0235 26590177 PMC4679089

[33] Um S Sopheab H . Breast and cervical cancer screening among women at reproductive age in Cambodia: A secondary analysis of Cambodia Demographic and Health Survey 2022. PloS One. (2025) 20:e0295881. doi: 10.1371/journal.pone.0295881 39883702 PMC11781645

[34] Aswathy S Quereshi MA Kurian B Leelamoni K . Cervical cancer screening: Current knowledge & practice among women in a rural population of Kerala, India. Indian J Med Res. (2012) 136:205–10. PMC3461731 22960886

[35] Sanavelli R Rao CVL Kothapally K . Awareness and acceptance of cervical cancer screening among women attending a tertiary care hospital: a cross-sectional study. Int J Reproduction Contraception Obstetrics Gynecology. (2026) 15:987–93. doi: 10.18203/2320-1770.ijrcog20260559

[36] Srivastava S Kurian K Garg PR Rehman A Garg R Rathi SK . Prevalence and predictors of cervical cancer screening among reproductive age group women: Evidence from cross-sectional study in Rohtak and Delhi. Asian Pac J Cancer Prev. (2022) 23:2771–7. doi: 10.31557/APJCP.2022.23.8.2771 36037133 PMC9741889

[37] Han CPP Soe HHK Than NN Lwin H Moe S . Prevalence and predictors of Pap-smear screening for cervical cancer among married women in urban of Mandalay, Myanmar. J Adv Med Med Res. (2016) 17:8. doi: 10.9734/BJMMR/2016/28156 31683997

[38] Shamaun S Jaleel R Gull Y Shahid A Iqbal M Qazi TN . Knowledge and attitude of cervical cancer screening and vaccination in patients attending gynecology outpatient clinic at a tertiary care hospital in Pakistan. South Asian J Cancer. (2023) 12:17–22. doi: 10.1055/s-0042-1751093 36851927 PMC9966166

[39] Hirani S Khan S Akram S Virji SN Shaikh PA Naeem E . Knowledge, awareness, and practices of cervical cancer, its risk factors, screening, and prevention among women in Karachi, Pakistan. Eur J Cancer Prev. (2021) 30:97–102. doi: 10.1097/CEJ.0000000000000590 32301762

[40] Imoto A Honda S Llamas-Clark EF . Human papillomavirus and cervical cancer knowledge, perceptions, and screening behavior: a cross-sectional community-based survey in rural Philippines. Asian Pac J Cancer Prev. (2020) 21:3145–51. doi: 10.31557/APJCP.2020.21.11.3145 33247669 PMC8033127

[41] Nilaweera RIW Perera S Paranagama N Anushyanthan AS . Knowledge and practices on breast and cervical cancer screening methods among female health care workers: a Sri Lankan experience. Asian Pac J Cancer Prev. (2012) 13:1193–6. doi: 10.7314/apjcp.2012.13.4.1193 22799304

[42] Witharana C Wijesiriwardhana P Jayasekara K Kumari P Rodrigo C . Awareness of female Malignancies among women and their partners in Southern Sri Lanka and implications for screening: a cross sectional study. BMC Public Health. (2015) 15:1179. doi: 10.1186/s12889-015-2531-6 26608133 PMC4660802

[43] Nguyen T-V Vu K-D Cu LTN Ngoc MHN Ho H-D . Socioeconomic inequalities in cervical cancer screening practices in Vietnam: a decomposition analysis. BMC Public Health. (2025) 25:1253. doi: 10.1186/s12889-025-22511-y 40181343 PMC11966878

[44] Phung MT An PL Vinh NN Le HHTC McLean K Meza R . A comparative study on behavior, awareness and belief about cervical cancer among rural and urban women in Vietnam. PloS Glob Public Health. (2023) 3:e0001817. doi: 10.1371/journal.pgph.0001817 37279208 PMC10243615

[45] International Agency for Research on Cancer . Cervical Cancer Screening (2022). Available online at: https://publications.iarc.who.int/Book-And-Report-Series/Iarc-Handbooks-Of-Cancer-Prevention/Cervical-Cancer-Screening-2022 (Accessed May 22, 2026).

[46] Stanczuk GA Baxter GJ Currie H Forson W Lawrence JR Cuschieri K . Defining optimal triage strategies for hrHPV screen-positive women-an evaluation of HPV 16/18 genotyping, cytology, and p16/Ki-67 cytoimmunochemistry. Cancer Epidemiol Biomarkers Prev. (2017) 26:1629–35. doi: 10.1158/1055-9965.EPI-17-0534 28887297

[47] Thrall MJ McCarthy E Mito JK Rao J . Triage options for positive high-risk HPV results from HPV-based cervical cancer screening: a review of the potential alternatives to Papanicolaou test cytology. J Am Soc Cytopathology. (2025) 14:11–22. doi: 10.1016/j.jasc.2024.09.003 39395892

[48] Arbyn M Smith SB Temin S Sultana F Castle P . Detecting cervical precancer and reaching underscreened women by using HPV testing on self samples: updated meta-analyses. BMJ. (2018) 363:k4823. doi: 10.1136/bmj.k4823 30518635 PMC6278587

[49] Ebrahimi N Yousefi Z Khosravi G Malayeri FE Golabi M Askarzadeh M . Human papillomavirus vaccination in low- and middle-income countries: progression, barriers, and future prospective. Front Immunol. (2023) 14:1150238. doi: 10.3389/fimmu.2023.1150238 37261366 PMC10227716

[50] Kabir SF Ashik-Ur-Rahman M Rahman A Hossen MA Rahman R Huq F . Factors associated with low utilization of cervical cancer screening services in Gazipur, Bangladesh. Obstet Gynecol Int. (2025) 2025:4476955. doi: 10.1155/ogi/4476955 41497355 PMC12767436

[51] Chatterjee S Chattopadhyay A Samanta L Panigrahi P . HPV and cervical cancer epidemiology - current status of HPV vaccination in India. Asian Pac J Cancer Prev. (2016) 17:3663–73. 27644600

[52] Salauddin M Irin F Disha P Ishi NS Akter S Ara R . Human papillomavirus in Bangladesh: Challenges and opportunities for prevention. Gynecol Oncol Rep. (2025) 59:101747. doi: 10.1016/j.gore.2025.101747 40309315 PMC12041753

[53] UNICEF . Pakistan Introduces HPV Vaccine to Protect Girls From Cervical Cancer, Joining 150 Countries (2025). Available online at: https://www.unicef.org/Pakistan/press-releases/Pakistan-introduces-hpv-vaccine-protect-girls-cervical-cancer-joining-150-countries (Accessed May 14, 2026).

[54] WHO . Accelerating Cervical Cancer Elimination Through HPV Vaccination in Nepal (2025). Available online at: https://www.who.int/news-room/feature-stories/detail/accelerating-cervical-cancer-elimination-through-hpv-vaccination-in-Nepal (Accessed May 14, 2026).

[55] Nazrul N Fouw M Beltman JJ Zeeuw J Schans J Koot J . Understanding cervical cancer awareness in hard-to-reach areas of Bangladesh: A cross-sectional study involving women and household decisionmakers. PloS One. (2024) 19:e0304396. doi: 10.1371/journal.pone.0304396 39121078 PMC11315347

[56] Rafi AA Orni KN Subah TA Akther S Acharjee A Zuhayr IA . Trends and burden of breast and cervical cancer in Bangladesh: A data-driven analysis and future outlook. (2025), 2025.12.05.25341679. doi: 10.64898/2025.12.05.25341679

[57] Interventions IWG on the E of C-P . Screen-and-treat approach and women at differential risk, in: Cervical Cancer Screening (2022). International Agency for Research on Cancer. Available online at: https://www.ncbi.nlm.nih.gov/books/NBK601994/ (Accessed July 25, 2026).

[58] Qiao L Li B Long M Wang X Wang A Zhang G . Accuracy of visual inspection with acetic acid and with Lugol’s iodine for cervical cancer screening: Meta-analysis. J Obstetrics Gynaecology Res. (2015) 41:1313–25. doi: 10.1111/jog.12732 26014371

[59] Ratyal S Saeed S Hameed N Akram S Khan MS Fatima A . Prevention and control of cervical cancer by WHO-endorsed guidelines for visual inspection with acetic acid (Via) as a simple screening method: Prevention and control of cervical cancer. Pakistan J Health Sci. (2024) 5:81–6. doi: 10.54393/pjhs.v5i12.2494

[60] Arbyn M Sankaranarayanan R Muwonge R Keita N Dolo A Mbalawa CG . Pooled analysis of the accuracy of five cervical cancer screening tests assessed in eleven studies in Africa and India. Int J Cancer. (2008) 123:153–60. doi: 10.1002/ijc.23489 18404671

[61] Kuhn L Saidu R Boa R Tergas A Moodley J Persing D . Clinical evaluation of modifications to a human papillomavirus assay to optimise its utility for cervical cancer screening in low-resource settings: a diagnostic accuracy study. Lancet Global Health. (2020) 8:e296–304. doi: 10.1016/S2214-109X(19)30527-3 31981559 PMC8720235

[62] Kelly H Jaafar I Chung M Michelow P Greene S Strickler H . Diagnostic accuracy of cervical cancer screening strategies for high-grade cervical intraepithelial neoplasia (CIN2+/CIN3+) among women living with HIV: A systematic review and meta-analysis. eClinicalMedicine. (2022) 53:101645. doi: 10.1016/j.eclinm.2022.101645 36187721 PMC9520209

[63] Goel B Desouza A Sehgal A Dubey S . Looking beyond VIA to improve cervical cancer screening in low resource settings. J Obstet Gynecol India. (2022) 72:503–8. doi: 10.1007/s13224-022-01674-3 36506901 PMC9732159

[64] Fokom Domgue J Dille I Gnangnon F Kapambwe S Bouchard C Mbatani N . Utility of colposcopy for the screening and management of cervical cancer in Africa: a cross-sectional analysis of providers’ training and practices. BMC Health Serv Res. (2024) 24:1619. doi: 10.1186/s12913-024-11982-1 39695604 PMC11658535

[65] Zhu J Norman I Elfgren K Gaberi V Hagmar B Hjerpe A . A comparison of liquid-based cytology and Pap smear as a screening method for cervical cancer. Oncol Rep. (2007) 18:157–60. doi: 10.3892/or.18.1.157 17549362

[66] Best SR Niparko KJ Pai SI . Biology of human papillomavirus infection and immune therapy for HPV-related head and neck cancers. Otolaryngol Clin North Am. (2012) 45:807–22. doi: 10.1016/j.otc.2012.04.005 22793854 PMC3398423

[67] Hernádi Z Szoke K Sápy T Krasznai ZT Soós G Veress G . Role of human papillomavirus (HPV) testing in the follow-up of patients after treatment for cervical precancerous lesions. Eur J Obstet Gynecol Reprod Biol. (2005) 118:229–34. doi: 10.1016/j.ejogrb.2004.06.029 15653209

[68] WHO . New Recommendations for Screening and Treatment to Prevent Cervical Cancer (2021). Available online at: https://www.who.int/news/item/06-07-2021-new-recommendations-for-screening-and-treatment-to-prevent-cervical-cancer (Accessed May 14, 2026).

[69] Buss LF Cury L Ribeiro CM Silva GAE Eluf Neto J . Access to colposcopy in the State of São Paulo, Brazil: probabilistic linkage study of administrative data. Cad Saude Publica. (2022) 38:e00304820. doi: 10.1590/0102-311X00304820 35043887

[70] Buss LF Levi JE Longatto-Filho A Cohen DD Cury L Martins TR . Attendance for diagnostic colposcopy among high-risk human papillomavirus positive women in a Brazilian feasibility study. Int J Gynaecol Obstet. (2021) 152:72–7. doi: 10.1002/ijgo.13362 33026115

[71] Maimela G Nene X Mvundla N Sawry S Smith T Rees H . The impact of decentralising colposcopy services from tertiary-level to primary-level care in inner-city Johannesburg, South Africa: a before and after study. BMJ Open. (2019) 9:e024726. doi: 10.1136/bmjopen-2018-024726 30928936 PMC6475219

[72] Benites-Zapata VA Hernandez-Bustamante EA Acuña-Chávez LM Escudero-Gaytan CP Ulloque-Badaracco JR Alarcón-Braga EA . Colposcopy in the primary health care: A scoping review. J Prim Care Community Health. (2023) 14:21501319231198942. doi: 10.1177/21501319231198942 37740513 PMC10517605

[73] Hull R Mbele M Makhafola T Hicks C Wang S-M Reis RM . Cervical cancer in low and middle-income countries. Oncol Lett. (2020) 20:2058–74. doi: 10.3892/ol.2020.11754 32782524 PMC7400218

[74] Dang L Kong L Zhao Y Dai Y Ma L Wei L . Evaluation of triage strategies for high-risk human papillomavirus-positive women in cervical cancer screening: A multicenter randomized controlled trial in different resource settings in China. Chin J Cancer Res. (2022) 34:496–509. doi: 10.21147/j.issn.1000-9604.2022.05.09 36398123 PMC9646459

[75] Blanckenberg ND Conradie HH Oettle CA Krige FK . Impact of the introduction of a colposcopy service in a rural South African sub-district on uptake of colposcopy. S Afr J OG. (2013) 19:81–5. doi: 10.7196/SAJOG.388 39041398

[76] Zeufack SCM Omoto J Owaya A Adoyo E Rop M Osongo CO . Feasibility of incorporating the Pocket colposcope into nurse-led cervical cancer screening programs in Western Kenya. Ecancermedicalscience. (2025) 19:1925. doi: 10.3332/ecancer.2025.1925 40606949 PMC12221255

[77] Lam CT Mueller J Asma B Asiedu M Krieger MS Chitalia R . An integrated strategy for improving contrast, durability, and portability of a Pocket Colposcope for cervical cancer screening and diagnosis. PloS One. (2018) 13:e0192530. doi: 10.1371/journal.pone.0192530 29425225 PMC5806883

[78] Catarino R Vassilakos P Scaringella S Undurraga-Malinverno M Meyer-Hamme U Ricard-Gauthier D . Smartphone use for cervical cancer screening in low-resource countries: A pilot study conducted in Madagascar. PloS One. (2015) 10:e0134309. doi: 10.1371/journal.pone.0134309 26222772 PMC4519052

[79] Asiedu MN Simhal A Chaudhary U Mueller JL Lam CT Schmitt JW . Development of algorithms for automated detection of cervical pre-cancers with a low-cost, point-of-care, Pocket Colposcope. IEEE Trans BioMed Eng. (2019) 66:2306–18. doi: 10.1109/TBME.2018.2887208 30575526 PMC6581620

[80] Ndapatani L Kubo E Munene A Odhiambo C Arodi S Marima R . Cervical cancer screening and treatment of VIA positive lesions among women living with HIV in Nairobi and Kajiado counties, Kenya: results from a targeted intervention implemented under the USAID Fahari ya Jamii project in supported HIV clinics. BMC Women’s Health. (2025) 25:621. doi: 10.1186/s12905-025-04185-1 41299522 PMC12750709

[81] Poli UR Bidinger PD Gowrishankar S . Visual inspection with acetic acid (VIA) screening program: 7 years experience in early detection of cervical cancer and pre-cancers in rural South India. Indian J Community Med. (2015) 40:203–7. doi: 10.4103/0970-0218.158873 26170547 PMC4478664

[82] Razzak MA Islam MN Aadeeb MS Tasnim T . Digital health interventions for cervical cancer care: A systematic review and future research opportunities. PloS One. (2023) 18:e0296015. doi: 10.1371/journal.pone.0296015 38100494 PMC10723694

[83] Mueller J Lam C Dahl D Asiedu M Krieger M Bellido-Fuentes Y . Portable Pocket colposcopy performs comparably to standard-of-care clinical colposcopy using acetic acid and Lugol’s iodine as contrast mediators: an investigational study in Peru. BJOG: Int J Obstetrics Gynaecology. (2018) 125:1321–9. doi: 10.1111/1471-0528.15326 29893472 PMC6115285

[84] Bae JK Roh H-J You JS Kim K Ahn Y Askaruly S . Quantitative screening of cervical cancers for low-resource settings: Pilot study of smartphone-based endoscopic visual inspection after acetic acid using machine learning techniques. JMIR mHealth uHealth. (2020) 8:e16467. doi: 10.2196/16467 32159521 PMC7097827

[85] Gallay C Girardet A Viviano M Catarino R Benski A-C Tran PL . Cervical cancer screening in low-resource settings: a smartphone image application as an alternative to colposcopy. Int J Womens Health. (2017) 9:455–61. doi: 10.2147/IJWH.S136351 28790867 PMC5489054

[86] Valls J Baena A Venegas G Celis M González M Sosa C . Performance of standardised colposcopy to detect cervical precancer and cancer for triage of women testing positive for human papillomavirus: results from the ESTAMPA multicentric screening study. Lancet Global Health. (2023) 11:e350–60. doi: 10.1016/S2214-109X(22)00545-9 36796982 PMC10020136

[87] Wentzensen N Massad LS Mayeaux EJ Khan MJ Waxman AG Einstein MH . Evidence-based consensus recommendations for colposcopy practice for cervical cancer prevention in the United States. J Low Genit Tract Dis. (2017) 21:216–22. doi: 10.1097/LGT.0000000000000322 28953109

[88] Xue P Ng MTA Qiao Y . The challenges of colposcopy for cervical cancer screening in LMICs and solutions by artificial intelligence. BMC Med. (2020) 18:169. doi: 10.1186/s12916-020-01613-x 32493320 PMC7271416

[89] Søfteland S Sebitloane MH Taylor M Roald BB Holmen S Galappaththi-Arachchige HN . A systematic review of handheld tools in lieu of colposcopy for cervical neoplasia and female genital schistosomiasis. Int J Gynaecol Obstet. (2021) 153:190–9. doi: 10.1002/ijgo.13538 33316096 PMC8248063

[90] Coole JB Brenes D Possati-Resende JC Antoniazzi M Fonseca BO Maker Y . Development of a multimodal mobile colposcope for real-time cervical cancer detection. BioMed Opt Express. (2022) 13:5116–30. doi: 10.1364/BOE.463253 36425643 PMC9664871

[91] Parham GP Mwanahamuntu MH Kapambwe S Muwonge R Bateman AC Blevins M . Population-level scale-up of cervical cancer prevention services in a low-resource setting: development, implementation, and evaluation of the cervical cancer prevention program in Zambia. PloS One. (2015) 10:e0122169. doi: 10.1371/journal.pone.0122169 25885821 PMC4401717

[92] Liu X Ning L Fan W Jia C Ge L . Electronic health interventions and cervical cancer screening: Systematic review and meta-analysis. J Med Internet Res. (2024) 26:e58066. doi: 10.2196/58066 39481096 PMC11565089

[93] Bagga R Singla R Srinivasan R Singh T Verma M . Experience with a novel community outreach approach to cervical cancer screening by colposcopy in the mobile unit. BMJ Innov. (2025) 11:246–251. doi: 10.1136/bmjinnov-2024-001277

[94] Romli R Abd Rahman R Chew KT Mohd Hashim S Mohamad EMW Mohammed Nawi A . Empirical investigation of e-health intervention in cervical cancer screening: A systematic literature review. PloS One. (2022) 17:e0273375. doi: 10.1371/journal.pone.0273375 35984812 PMC9390916

[95] Firnhaber C Mao L Levin S Faesen M Lewis DA Goeieman BJ . Evaluation of a cervicography-based program to ensure quality of visual inspection of the cervix in HIV-infected women in Johannesburg, South Africa. J Low Genit Tract Dis. (2015) 19:7–11. doi: 10.1097/LGT.0000000000000040 24914887 PMC4272220

[96] Shamsunder S Digumarti L Nayak B Dasari V Mishra A Kumar A . Bringing cervical cancer screening closer to women: Feasibility of artificial intelligence and remote assessment in primary health care. Int J Public Health. (2026) 71:1609094. doi: 10.3389/ijph.2026.1609094 41867352 PMC12999539

[97] Craig A Lawford H Miller M Chen-Cao L Woods L Liaw S-T . Use of technology to support health care providers delivering care in low- and lower-middle-income countries: Systematic umbrella review. J Med Internet Res. (2025) 27:e66288. doi: 10.2196/66288 40533075 PMC12223456

